# β Cell Dysfunction Versus Insulin Resistance in the Pathogenesis of Type 2 Diabetes in East Asians

**DOI:** 10.1007/s11892-015-0602-9

**Published:** 2015-05-06

**Authors:** Daisuke Yabe, Yutaka Seino, Mitsuo Fukushima, Susumu Seino

**Affiliations:** Center for Diabetes, Endocrinology and Metabolism, Kansai Electric Power Hospital, 2-1-7 Fukushima-ku, Osaka, 553-0003 Japan; Center for Metabolism and Clinical Nutrition, Kansai Electric Power Hospital, 2-1-7 Fukushima-ku, Osaka, 553-0003 Japan; Division of Molecular and Metabolic Medicine, Kobe University Graduate School of Medicine, Chuo-ku Kobe, 650-0017 Japan; Department of Nutritional Science, Okayama Prefectural University, 111 kuboki, Soujiya-shi, Okayama, 719-1197 Japan

**Keywords:** Type 2 diabetes, Insulin secretion, Insulin resistance, East Asian, Incretin

## Abstract

Type 2 diabetes (T2DM) is one of the most serious global health problems and is mainly a result of the drastic increase in East Asia, which includes over a fourth of the global diabetes population. Lifestyle factors and ethnicity are two determinants in the etiology of T2DM, and lifestyle changes such as higher fat intake and less physical activity link readily to T2DM in East Asians. It is widely recognized that T2DM in East Asians is characterized primarily by β cell dysfunction, which is evident immediately after ingestion of glucose or meal, and less adiposity compared to the disease in Caucasians. These pathophysiological differences have an important impact on therapeutic approaches. Here, we revisit the pathogenesis of T2DM in light of β cell dysfunction versus insulin resistance in East Asians and discuss ethnic differences in the contributions of insulin secretion and insulin resistance, together with incretin secretin and action, to glucose intolerance.

## Introduction

The rapidly increasing type 2 diabetes (T2DM) worldwide is one of the most serious health problems today. The number of patients with diabetes, estimated to be 385 million in 2014, is expected to be 592 million by 2035 [1], partly due to the drastic increase of patients in East Asian countries, which now includes a fourth of the global diabetes population (Table 1). T2DM in East Asian countries are characterized by lesser obesity and younger age of onset [2, 3] It is known that lifestyle factors and ethnicity are important determinants in the etiology of T2DM [4, 5••], and it is suggested that lifestyle changes such as higher fat intake and less physical activity link more readily to T2DM development in East Asians, as first demonstrated in studies of Japanese Americans in comparison with native Japanese counterparts [6, 7•]. To prevent and better manage T2DM in East Asian countries, it is important to understand the pathophysiology of T2DM in East Asians.

**Table 1:** Comparison of prevalence of diabetes, proportion undiagnosed, age distribution, and related expenditure among adults aged 20–79 in East Asian countries compared to the USA

	Prevalence (%)	Cases	% undiagnosed	% aged 20–39	% aged 60–79	Cost per case (USD)
World	8.3	386,667,276	46.3	17.2	34.9	1583
United States of America	5.4	25,779,345	27.7	12.0	45.6	4466
China	9.3	96,288,029	53.3	13.8	36.9	421
Hong Kong	9.9	568,384	54.0	5.2	50.7	1811
Macao	9.3	43,080	54.0	7.5	40.4	1027
Japan	7.6	7,212,052	54.0	5.1	62.0	4908
Dem. People’s Republic of Korea	6.7	1,163,480	63.0	13.8	32.5	n.a.
Republic of Korea	7.3	2,767,693	54.0	6.5	46.4	2144
Mongolia	7.3	133,754	53.3	42.0	9.2	337
Taiwan	9.9	1,757,050	54.0	8.5	47.3	1219

Data source [1]

*Dem.* Democratic, *n.a.* not available

T2DM in East Asians is characterized primarily by β cell dysfunction, which is evident immediately after ingestion of glucose or mixed meal, and less obesity compared to Caucasians [8•, 9•, 10]. Insulin resistance, as indicated by the homeostatic model assessment (HOMA) of insulin resistance (IR), is generally higher in Caucasians, while β cell response, as measured by HOMA of β cell function and insulinogenic index (IGI), is lower in East Asians. These pathophysiological differences in the manifestation of the disease have a crucial impact on the appropriate preventive and therapeutic approaches. In this article, we revisit the pathogenesis of T2DM with reference to β cell dysfunction versus insulin resistance, together with incretin secretion and action, in East Asians and discuss ethnic differences in the contributions of insulin secretion and insulin resistance to glucose intolerance.

## Rapid Increase in T2DM and Westernized Lifestyle Changes Among East Asians

Historically, the prevalence of T2DM among East Asians was low compared with that in the United States of America (USA). Nevertheless, reports of a higher diabetes prevalence in Japanese Americans than in the general American population indicated that Japanese are not protected from diabetes. Indeed, in the early 1960s, the prevalence of diabetes in Hawaii was found to be 20.1 per 1000 person-years for Japanese and 7.3 for Caucasian [11], suggesting that Japanese might be at a special risk of developing diabetes upon exposure to lifestyles in the USA. This notion was further supported by research demonstrating higher rates of glucose intolerance among Japanese Americans living in Hawaii and Los Angeles than that in native Japanese [12]. About the same time, West found that lifestyle-related factors and obesity exert an especially strong influence on the progression of diabetes in Native Americans [13]. It is now widely accepted that obesity, through its association with insulin resistance, increases the risk of T2DM [14]. Japanese Americans, with lower body mass index (BMI) compared to other ethnic groups, develop diabetes at a rate that is more often associated with obesity in Caucasian [15]. In the late 1970s, Fujimoto et al. initiated the study of Japanese Americans in Seattle to clarify why Japanese Americans so readily develop diabetes [7•, 16]. They reported that daily calorie intake was comparable between Japanese Americans and native Japanese although less than that of Caucasians, but that Japanese Americans consumed fats in amounts similar to that of Caucasians, which were in fact much higher than that of native Japanese. Thus, Japanese Americans who adopted western dietary habits including higher consumption of animal fat showed higher rates of diabetes. The apparent high sensitivity of Japanese Americans to western dietary habits in terms of diabetes development required further investigation on pathophysiology of T2DM of East Asians.

## Insulin Secretion and Resistance in East Asians

T2DM is characterized by insulin resistance and impaired insulin secretion. It has been proposed, based mainly on studies of Caucasian subjects, that T2DM is triggered by insulin resistance, which is compensated initially by increased β cell response, which eventually leads to T2DM due to exhaustion of pancreatic β cells [17–19]. However, as reported by our group and others, Japanese pre-diabetes and early-stage diabetes are both characterized by reduced insulin secretion along with lower insulin resistance when compared to Caucasians [20–23]. These studies indicate profound differences in T2DM pathophysiology of East Asians that may be relevant for prevention and treatment of diabetes in East Asian countries.

**Fig. 1:**
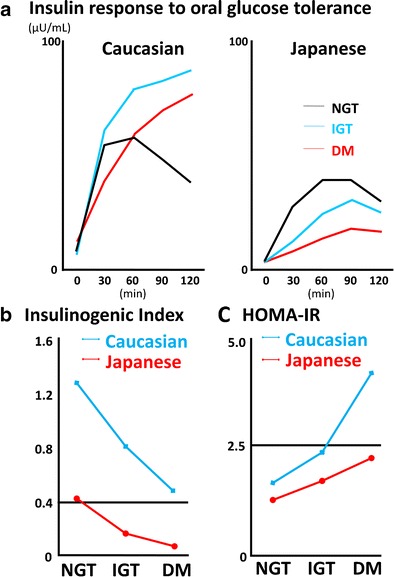
Insulin secretion and insulin resistance in Caucasian and Japanese. **a** Comparison of insulin response to a 75-g glucose tolerance test in Caucasian and Japanese with normal glucose tolerance (NGT), impaired glucose tolerance (IGT), and type 2 diabetes (DM). **b** Comparison of insulinogenic index in Caucasian and Japanese with NGT, IGT, and DM. **c** Comparison of homeostatic assessment insulin resistance (HOMA-IR) in Caucasian and Japanese with NGT, IGT, and DM (reproduced with permission from [10])

Insulin secretory capacity has been well characterized by HOMA-β and IGI during the oral glucose tolerance test (OGTT) and, to a lesser degree, by acute insulin response during intravenous glucose tolerance test (IVGTT). Our previous studies as early as the 1970s indicated that the insulin response to ingestion of glucose in Japanese, both in normal glucose tolerance (NGT) and T2DM, was much lower than that in Caucasian [24–27]. Later, cross-sectional studies in Japanese subjects with NGT, impaired glucose tolerance (IGT), and T2DM confirmed reduced insulin secretion in Japanese in comparison to those of Caucasian (Fig. 1) [20, 28]. These studies suggest that Japanese may be characterized by impaired early-phase insulin secretion, as the IGI of Japanese is lower throughout NGT via IGT to T2DM (Fig. 1), while the IGI is higher in Caucasian than in Japanese throughout all of the stages of glucose tolerance [20, 28]. Reduced insulin secretory capacity, especially in the early phase, has been reported not only in Japanese but also in other East Asians such as Korean [29, 30] and Chinese [31, 32]. Our previous investigations also suggested that the acute insulin response during IVGTT was substantially lower in Japanese compared to Caucasian [33, 34]. These findings are supported by recent important studies: (1) systematic review and meta-analysis of insulin response to glucose in IVGTT revealing reduced insulin secretory capacity of East Asians compared to Caucasians and Africans [35] and (2) studies on matched cohorts of Caucasian and Japanese subjects in OGTT and IVGTT revealing reduced β cell function in Japanese [8•, 9•]. Thus, a reduced capacity of insulin secretion is typical of East Asians, which could render them sensitive to the development of diabetes in conditions of over-nutrition.

Insulin resistance is usually evaluated by HOMA-IR, to a lesser degree by Matsuda index or ISI composite calculated by OGTT data and less by *S*_I_, an index of insulin sensitivity derived from IVGTT data calculated by minimal model analysis. Cross-sectional studies in Japanese subjects with NGT, IGT, and T2DM demonstrated that HOMA-IR is lower in Japanese throughout NGT, IGT, and T2DM in comparison with that of Caucasian in the Botnia Study (Fig. 1) [20, 28]. In addition, Tripathy et al. reported that HOMA-IR increased approximately 2-fold as glucose tolerance deteriorated from NGT to IGT, and 3.6-fold from NGT to T2DM [28], but the change in HOMA-IR in Japanese from NGT via IGT to T2DM is not so drastic [20]. Our previous investigation using minimal model analysis during IVGTT also revealed more preserved insulin sensitivity in Japanese T2DM patients when compared indirectly to Caucasian T2DM patients [33, 34]. These findings are supported by recent studies: (1) systematic review and meta-analysis of insulin sensitivity in IVGTT finding less insulin resistance in East Asians compared to Caucasians and Africans [35] and (2) studies on matched cohorts of Caucasian and Japanese subjects revealing lower HOMA-IR and Matsuda index, but not higher *S*_I_, in Japanese throughout the different stages of glucose tolerance [8•, 9•].

Counterbalance between insulin secretion and insulin resistance is critical for T2DM pathogenesis. Being less obese with less insulin resistance, Japanese have a greater amount of visceral fats in comparison with Caucasians after adjusting for age, gender, and subcutaneous fats [36], suggesting that Japanese easily accumulate visceral fats. Thus, a subtle increase in insulin resistance due to visceral fat accumulation might well disturb the fine balance with the reduced insulin secretory capacity often seen in East Asians and easily trigger onset of type 2 diabetes. This model might well explain why there is a higher proportion of isolated IGT in isolated impaired fasting glucose (IFG), IFG/IGT, and isolated IGT in Asia than that found in Europe [37, 38]. We previously demonstrated in Japanese that the IGI is significantly reduced from NGT via isolated IGT to T2DM, while HOMA-IR is slightly but not significantly increased from isolated IGT to T2DM [39, 40]. In addition, we also demonstrated that HOMA-IR is significantly increased from NGT via isolated IFG to T2DM along with a significant reduction in IGI [40, 41]. While conversion rates from NGT to T2DM via isolated IGT, IFG/IGT, and isolated IGT need to be compared between Asians and Caucasians prospectively in the future, the model also underlie the appearance of diabetes in East Asians when not nearly as obese as Caucasian [5••, 42] as well as the increased T2DM prevalence among East Asian people such as Japanese Americans in the USA who consume similar daily energy but significantly more fats and less carbohydrates than their counterparts in Japan [43, 44]. This is consistent with the fact that diabetes is rapidly increasing in Japan and across East Asian countries today along with reduced intake of carbohydrates and increased intake of animal fats (Table 2). Future prospective studies are also needed to relate changes in dietary pattern with changes in indices related to insulin secretion and insulin resistance, along with visceral fat accumulation and adiposity-related indices including adiponectin, TNF-alpha, and newly identified progranulin [45], during the development of T2DM in East Asians.

**Table 2:** Changes in dietary pattern in East Asian countries and United States of America

Japan	Year of survey	1950	1960	1970	1980	1990	2000	2005	2010
	Total energy intake (kcal)	2098	2096	2210	2219	2026	1948	1904	1849
	Protein (%)	13.0	13.3	14.0	14.2	15.5	16.0	16.2	14.6
	Fat (%)	7.7	10.4	18.9	22.6	25.3	26.5	25.1	26.1
	Carbohydrate (%)	79.7	76.1	66.6	55.7	56.7	54.6	56.1	55.7
China	Year of survey	1952	1962	1970	1982	1992	2000	2004	2009
	Total energy intake (kcal)	2056	1697	1978	2518	2328	M2146/F1941	M2064/F1807	M1943/F1969
	Protein (%)	9.3	9.7	9.6	10.6	11.7	M24.0/F23.7	M24.6/F24.4	M25.5/F24.4
	Fat (%)	7.6	5.5	7.4	17.5	22.5	M26.3/F26.4	M26.9/F26.4	M27.8/F29.2
	Carbohydrate (%)	83.0	84.8	82.9	71.8	65.8	M58.9/F58.7	M57.8/F58.3	M56.2/F54.9
Korea	Year of survey			1969	1979	1989	2000	2005	2010
	Total energy intake (kcal)			2105	2098	1871	1863	1826	1691
	Protein (%)			12.5	13.3	16.1	16.4	16.6	14.7
	Fat (%)			7.2	11.2	13.4	19.7	21.3	20.0
	Carbohydrate (%)			80.4	75.3	69.1	63.9	62.1	65.1
United States of America	Year of survey	1950	1960	1970	1980	1990	2000	2005	2010
	Total energy intake (kcal)	3200	3100	3300	3500	3800	4200	4100	4000
	Protein (%)	11.8	11.9	11.9	12.7	12.4	11.8	12.0	12.0
	Fat (%)	39.1	40.1	40.1	41.7	39.6	40.9	42.6	42.8
	Carbohydrate (%)	52.0	50.1	48.6	46.6	49.3	48.1	47.6	47.4

Data source: Japan, The National Health and Nutrition Survey, Japan and The National Nutrition Survey, Japan; China, Eur J Clin Nutr. 1993;47(5):333–46 and British Journal of Nutrition (2012), 108, 1292–1299; Korea, Malays Journal of Nutrition. 2003; 9(1):7–17 and Nutrition Research 2014; 34: 760–770; Unites States of America, U.S. Department of Agriculture, Center for Nutrition Policy and Promotion, Nutrient Content of the U.S. Food Supply. *M* male, *F* female

## Unsolved Questions Regarding Low Insulin Secretory Capacity in East Asians

An important question is why East Asians show a low reserve capacity of insulin secretion. Genome-wide association study (GWAS) in Japanese found that variants in *KCNQ1*, the potassium voltage-gated channel subfamily Q member 1 gene, associates with T2DM [46, 47]. The KCNQ1 protein is expressed in insulin-secreting cultured cells, and the KCNQ1 blocker inhibits tolbutamide-stimulated insulin secretion, which suggests involvement of KCNQ1 in insulin secretion [48]. Following identification of variations in the *KCNQ1* gene, GWAS identified in Japanese genetic variants associated with T2DM near the *UBE2E2* gene and the *C2CD4A/B* gene [49]. The *UBE2E2* gene encodes the ubiquitin-conjugating enzyme E2E2 expressed in the human pancreas and cultured insulin-secreting cells and is implicated in normal biosynthesis and secretion of insulin in pancreatic β cells [50]. The *C2CD4A/B* gene showed expression profiles similar to that of the *UBE2E2* gene, but its protein function remains largely unknown. GWAS identified in Taiwanese T2DM-associated variants at the *PTPRD* and *SRR* loci [51] as well as the *SPRY2* and *CDC123* loci [52]. The *PTPRD* gene belongs to the receptor type IIA subfamily of protein tyrosine phosphatases, which has been implicated in neural development, cancer, and diabetes, but its function is also obscure. The *SRR* gene encodes a serine racemase that synthesizes d-serine from l-serine; dysregulation of d-serine could affect insulin secretion in the pathogenesis of T2DM [53, 54]. The *SPRY2* and *CDC123* genes encode proteins of the sprout family and the Ca^2+^/calmodulin-dependent protein kinase 1 subfamily, but their roles in T2DM development are unknown. More recently, studies identified several additional T2DM-susceptible genes in East Asians [55, 56], but it is difficult at this stage to know how these genes might affect β cell function in East Asians.

While many T2DM-susceptible genes found in GWAS seem related to β cell function, whether or not genetic variants in these loci might explain the reduced insulin secretory is dubious. As mentioned above, the association of westernized high fat dietary and more sedentary lifestyle habits along with the rapid increase of T2DM in East Asia suggests a thrifty gene hypothesis by which T2DM is caused by genetic variants undergoing positive selection during historical times of nutrient scarcity [57]. This hypothesis was tested for 17 confirmed T2DM-susceptible loci as well as 15 loci identified in East Asians, but no consistent selection patterns were detected [58, 59]. In addition, most of these T2DM-susceptible genes are replicated in non-East Asians, demonstrating that none of the variants itself can explain the reduced β cell function that is characteristic of East Asians. Future studies of gene-environment interactions, gene-gene interactions, and epigenetic modifications are definitely required to clarify the unique pathophysiology of East Asian diabetes.

## Incretin as a Possible Link to β Cell Dysfunction in East Asians

Incretin is an important area of research related to β cell function; it has been demonstrated that incretins are responsible for more than 50–70 % of post-challenged insulin secretion in Caucasian [60–62]. The incretins, glucose-dependent insulinotropic polypeptide (GIP) and glucagon-like peptide-1 (GLP-1), are secreted from the gut in response to ingestion of various nutrients including carbohydrates, proteins, and lipids, and enhance insulin secretion glucose dependently to exert their glucose-lowering effects [63, 64]. While earlier studies reported reduced GLP-1 secretion and enhanced GIP secretion in Caucasian T2DM [65, 66•, 67], later studies failed to confirm this [68•, 69•, 70], which strongly suggests that incretin secretion per se is not involved in the pathogenesis of T2DM in Caucasian. Recently, our group and others have characterized secretions of GLP-1 and GIP among NGT and T2DM and found that there are no differences among the two groups in Japanese [23, 71–73] or Korean [74, 75], indicating that incretin secretion per se is not involved in the pathogenesis of T2DM in East Asians, similar to the case in Caucasian. However, it is noteworthy that meal-induced secretion of GLP-1 is negligible in Japanese [23, 71, 76] and that the GLP-1 secretion in repose to 75-g OGTT is lower in Japanese compared to that of Caucasian when measured by the same assay system [23, 71, 77]. It remains to be determined whether the difference in GLP-1 secretion contributes to a difference in β cell function between East Asians and Caucasian.

Another difference that might contribute to ethnic variance in insulin secretory capacity could be impaired incretin action, which is a major pathophysiological characteristic of T2DM [60–62]. Attenuated GIP-induced but not GLP-1-induced insulin secretion is thought to play a role [78]. It has been shown that genetic variants of the GIP receptor have been identified as T2DM-susceptible genes by GWAS, confirming the importance of incretins in T2DM progression [79]. However, it has been demonstrated recently that the incretin effect is not impaired in Japanese and Korean T2DM subjects [75, 80]. We recently found a novel mechanism underlying glucose-dependent insulinotropic action of incretins, which is impaired in obese model rats but not in non-obese diabetic model rats [81], which is consistent with an impaired incretin effect in Caucasian and near-normal incretin effect in East Asians. As studies of incretin action on glucose-induced insulin secretion in East Asians are limited, further investigations are required to determine whether or not incretin action is involved in reduced insulin secretory capacity in East Asians.

## Therapeutics for Better Prevention and Management of T2DM in East Asians

Due to reduced β cell function and lower insulin resistance, insulin secretagogues such as sulfonylureas (SU) and glinides or insulin injections have been used in the management of T2DM in Japan. In contrast to their superior effects in T2DM of Japanese and East Asians, SU and glinides are associated with hypoglycemia and body weight gain. Recently, incretin-based therapies, dipeptidyl peptidase-4 inhibitors (DPP-4i) and glucagon-like peptide-1 receptor agonists (GLP-1RA), have become widely available for management of type 2 diabetes and are used especially in East Asia. Although involvement of incretins in β cell dysfunction in East Asians is largely unknown as mentioned above, recent meta-analyses of clinical trials on DPP-4i and GLP-1RA found that the drugs are more effective in Asians [82, 83•, 84•]. Incretin-based therapies exert their glucose-lowering effects most likely by ameliorating primary β cell dysfunction through increased incretin activity [85, 86]. The greater HbA1c-lowering effects of incretin-based therapies in East Asians may confirm that β cell dysfunction has a greater responsibility for hyperglycemia in East Asians, compared with Caucasian.

Our recent studies have found a possible link between dietary habits and the efficacy of DPP-4i. The HbA1c-lowering effects of DPP-4i are enhanced by fish intake, as estimated by food records and serum levels of eicosapentaenoic acids and docosahexaenoic acids, in Japanese T2DM patients [87••, 88], presumably because nutrients in fish promote GLP-1 secretion. Indeed, we demonstrated in a cross-over setting that eating fish before rice enhanced GLP-1 secretion and ameliorated postprandial glucose excursions by increasing insulin secretion and delaying gastric emptying, in comparison with those eating fish after rice (manuscript in preparation) [89]. Similar reversal of rice and meat, which is rich in saturated and mono-unsaturated fats that enhance not only GLP-1 secretion but also that of GIP, fails to ameliorate glucose excursions may possibly facilitate fat accumulation. Indeed, small but significant body weight gain, presumably due to enhanced GIP secretion by consumption of saturated and mono-unsaturated fatty acids and increased fat deposition by GIP and these fats, is associated with deterioration of the HbA1c-lowering effects of DPP-4i in Japanese T2DM patients [90, 91]. Thus, the greater efficacy of DPP-4i in Asians may be partly due to dietary habits along with lesser insulin resistance and adiposity.

Greater glucose-lowering effects and low hypoglycemic risk of DPP-4i in Asian T2DM have made DPP-4i a widely used drug in non-obese T2DM in East Asia, especially in Japan [92, 93]; metformin serves as a first-line drug in obese T2DM in Caucasian [94, 95]. Although incretin-based therapies are thought to ameliorate β cell dysfunction with little hypoglycemia risk, cases of severe hypoglycemia were reported when DPP-4i was first introduced in Japan [96•]. The estimated incidence of hypoglycemic coma with DPP-4i sitagliptin was 16.3 per million patients during the first 6 months after its launch, approximately 6.4-fold higher in Japan than that in the USA in the corresponding period. This was partly due to the use of DPP-4i with SU, which had been widely used to improve β cell dysfunction in Japan [93]. Recent studies have detailed the mechanisms underlying severe hypoglycemia with DPP-4i and SU combinations [97–100]; careful consideration to dose-titration and patient education when initiating this combination can avoid this outcome as well as improve β cell dysfunction characteristic of T2DM in East Asians.

## Conclusion

T2DM of East Asians is characterized by β cell dysfunction rather than insulin resistance due to increased adiposity, which requires a preventative and therapeutic approach that targets β cell dysfunction precisely. While it presently remains unclear why East Asians have reduced insulin secretory capacity, lifestyle and pharmacological interventions should be rigorously investigated in the future for better prevention and management of T2DM in East Asia.
